# Policy Series: Ageism: Outcomes, Interventions, Future Directions

**DOI:** 10.1093/geroni/igab046.1665

**Published:** 2021-12-17

**Authors:** Kelly Trevino, Becca Levy

## Abstract

Ageism is stereotyping and discrimination against individuals or groups based on their age. Ageism toward older adults is ubiquitous in American society and takes many forms including prejudicial practices and institutional policies that lead to unfair treatment of older adults. Ageism negatively impacts older adults in numerous domains such as health care and the workplace. Older adults themselves often internalize ageist views with detrimental effects on physical and mental health including increased risk for suicidal ideation and worse memory performance. This symposium addresses ageism from multiple perspectives and describes strategies for detecting and combatting ageism. The first speaker is Patricia D’Antonio, Vice President of Policy and Professional Affairs of The Gerontological Society of America (GSA). Ms. D’Antonio will describe GSA’s Reframing Aging Initiative which aims to improve the public’s understanding of the meaning of aging in order to counter ageism and support policies and programs that benefit older adults. The second speaker, Dr. Fredriksen Goldsen will use an Age Equity Framework to present her research on the relationship between ageism and mental and physical health and quality of life in LGBTQ older adults. The third speaker, Dr. Gendron will describe a content analysis of an anti-ageism resource that evaluates ageism interventions using an ecological framework. The fourth speaker, Dr. Hinrichsen will discuss ways psychotherapists can help older adults identify and move beyond internalized ageist beliefs. Finally, Dr. Levy, an internationally recognized expert in ageism will discuss themes across speakers and comment on the future of work in this area.

